# Association Between Dietary Protein Sources and Nutrient Intake in the Diet of Canadian Children

**DOI:** 10.3390/nu17111834

**Published:** 2025-05-28

**Authors:** Hrvoje Fabek, Shekoufeh Salamat, G. Harvey Anderson

**Affiliations:** Department of Nutritional Sciences, Temerty Faculty of Medicine, University of Toronto, Toronto, ON M5S 1A8, Canada; hrvoje.fabek@utoronto.ca (H.F.); s.salamat@utoronto.ca (S.S.)

**Keywords:** dietary protein, Canadian Community Health Survey, protein quality, plant-based protein, animal protein, nutrient adequacy, food intakes, children

## Abstract

Background/Objectives: Canada’s 2019 Food Guide (CFG) encourages the increased consumption of plant-based foods as dietary protein sources. However, the nutritional implications of replacing animal-based proteins with plant-based alternatives in children’s diets remain unclear. This study aimed to examine the association between protein food sources and nutrient intake in Canadian children aged 9–18 years. Methods: We analyzed data from 2324 children from the 2015 Canadian Community Health Survey (CCHS), using the Public-Use Microdata File (PUMF) containing 24 h dietary recalls. Participants were categorized into four groups based on the proportion of protein from plant sources: Group 1 (0–24.9%), Group 2 (25–49.9%), Group 3 (50–74.9%), and Group 4 (75–100%). Nutrient intakes were compared and assessed against the Recommended Dietary Allowances (RDAs) and Adequate Intake (AI). Results: Groups 1 and 3 had less favorable macronutrient profiles than Group 2. A 3:1 animal-to-plant protein ratio (Group 2) aligned most closely with dietary recommendations. Groups 1 and 2 exceeded RDAs for protein, iron, vitamin B12, thiamine, riboflavin, niacin, vitamin B6, and zinc by over 146% (about four SDs above the mean requirement), suggesting a low risk of inadequacy, although saturated fat intake was high. The intakes of vitamin D and folate were below 66% of the RDA, while calcium and magnesium were below 100% in some subgroups, with probabilities of inadequacy of 0.93 and 0.31, respectively. Group 4 (2.71%) was too small for reliable analysis. Conclusions: An approximate 3:1 ratio of animal-to-plant protein sources may represent an optimal balance for supporting nutrient intake and improving macronutrient profiles in the diets of Canadian children.

## 1. Introduction

The relationship between diet and chronic diseases is well-established and begins in childhood or early adolescence [1], and their dietary patterns are often maintained into adulthood [2]. The consumption of fruits, vegetables, whole grains, and dairy products in Canadian children during school hours was found to be below recommendations, reflecting below-average diet quality [3] and a high level of foods to limit [4]. Health Canada has utilized the Eating Right campaign to emphasize greater intakes of plant foods, consistent with the 2019 Canada’s Food Guide (CFG) [5].

The 2019 CFG focuses on the prevention of chronic disease, rather than the past emphasis on serving sizes and nutrients. The CFG offers a snapshot of a plate illustrating the relative proportions of foods to consume. It emphasizes choosing proteins from plants more often [5]. Although the impact of the new CFG on public health remains to be seen, recent evidence shows that the dietary pattern depicted in the 2019 CFG Snapshot appears to be inadequate for meeting the calcium and vitamin D needs of children and adolescents [6]. Our recent examination of the 2015 Canadian Community Health Survey (CCHS) showed that increasing the consumption of plant protein foods resulted in reduced intakes of many nutrients, including protein, vitamin D, calcium, vitamin B12, and zinc in adults, with greater impact on the elderly [7]. Similarly, another evaluation of the protein intake of Canadian adults concluded that older adults and females are at risk of not meeting protein and other nutrient recommendations [8]. However, the impact of decreasing the animal-to-plant protein ratio on the nutritional quality of Canadian children’s diets has not been described.

The present study aims to address this gap by examining the relationship between protein food sources and nutrient intakes in the diets of Canadian children aged 9–18 years using data from the 2015 CCHS. This study provides insights into the nutritional implications of varying animal-to-plant protein ratios in children’s diets, and suggests a ratio of 3:1.

## 2. Materials and Methods

### 2.1. Data Source and Study Population

Data from the CCHS 2015 Public-Use Microdata File (PUMF) were used for this study, and the methodology employed reported on adults aged 19 and older [7]. The CCHS 2015 is a voluntary, nationally representative survey conducted by Statistics Canada. This survey has a cross-sectional design with three sampling stages. Interviews were computer-assisted using a 5-step automated multi-pass method (AMPM) adapted and modified for the Canadian population from the United States Department of Agriculture (USDA), and were conducted between 2 January 2015 and 31 December 2015, on all days of the week. Two separate questionnaires, a (a) 24 h dietary recall and (b) a detailed support document to complement the 24 h dietary recall, were administered. This information, collected by Statistics Canada, provides the most current nutrition data available for Canadians. The survey is representative of twelve age–sex groups, which correspond to the Dietary Reference Intakes (DRI) groupings [9]. The final sample of the CCHS included 20,487 respondents, with a 61.6% response rate. During the first interview, a random subset of 35% of the initial respondents was asked to complete a second 24 h dietary recall over the phone within 3–10 days of the first interview.

Detailed information on the sampling of respondents can be found in the CCHS, Nutrition user guide [10]. The CCHS 2015 collected 24 h food recalls of Canadian children, providing an opportunity to evaluate their food and nutrient intakes. The CCHS 2015 obtained 24 h dietary recalls across all seasons and days of the week to ensure the regional representativeness of dietary intake.

Because the 2019 CFG recommended increasing the intake of plant foods and decreasing the intake of animal food sources, we categorized the survey results into four groups, reflecting an increasing ratio of plant foods in the diet. For this report, the single 24 h dietary recalls of males and females aged 9 to 18 years were utilized. The final sample consisted of *n* = 2324 participants, with 1151 females and 1173 males.

### 2.2. Adjusting for Misreporting

Analysis was carried out on all plausible reporters as identified by statistics Canada, which accounted for the over- and under-reporting of energy intake and total energy expenditure (TEE) for each respondent as reported by [11]. The Institute of Medicine’s equations were used to predict TEE based on age, sex, height, weight, and physical activity levels (i.e., sedentary, low active, moderately active and highly active) when measured height and weight or self-reported height and weight were available in the dataset. Reported energy intakes (EI) were compared with each respondents’ TEE to identify misreporters. Under- and over-reporting were defined as the ratio of EI:TEE < 0.7 and >1.42, respectively, whereas those in between were considered plausible reporters. Children’s (9 to 18 years of age) self-reported heights and weights were adjusted according to the correction factor applied by Statistics Canada [12].

BMI categories were based on standard classifications for children and adolescents [13]. To categorize physical activity level in 2015, respondents’ average physical activity per day in minutes was computed by dividing the variable PHSGAPA (hours of physical activity per week) by 7 and multiplying by 60. Cut-offs to define sedentary, low active, active, and very active were then applied from Health Canada’s Reference Guide to Understanding and Using the Data for CCHS 2015 [10], whereby underweight respondents (9 to 18 years of age with a BMI < 18.5 kg/m^2^) were excluded.

### 2.3. Definition of Protein Groups

Increasing patterns of plant protein consumption were used to evaluate the nutrient intakes reported in the 24 h food recalls of 9- to 18-year-olds. Respondents were allocated into one of four groups, defined based on their protein intake from plant-derived food sources: Group 1 (0–24.9%), Group 2 (25–49.9%), Group 3 (50–74.9%), and Group 4 (75–100%). The plant-to-total protein intake ratio categories (0–24.9%, 25–49.9%, 50–74.9%, and 75–100%) were established a priori to represent gradations of plant protein intake aligned with recognizable dietary patterns, rather than being based on statistical distributions of the population based on quartiles. The 25–49.9% category, which reflected the most common intake range for the population, was selected as the reference group due to its relevance and sample size.

The Canadian Nutrient File (CNF) contains a comprehensive database of the nutritional composition of generic foods found in the Canadian marketplace.

### 2.4. Nutrient Intakes Analysis

Nutrient intakes, in macro- and micronutrient amounts (g, mg, or μg/day), were identified within each of the 4 groups. Group 2, comprising 25–49.9% of the respondents, was designated as the reference group. Intakes from each of the 3 groups were compared to the reference group.

The average daily nutrient intakes are compared among groups and in relation to the Recommended Dietary Allowances (RDA) or Adequate Intake (AI) for 9- to 13-year-old females and males, and 14- to 18-year-old females and males. Additionally, the average intakes are expressed as a percentage of the RDAs and the AI. However, this does not account for the expected variability in individual requirements, as well as the variation in intake among individuals, and leads to an overestimation of the incidence of inadequate intakes [14]. Therefore, the probability approach described by Anderson [15] and Beaton [16,17] was applied to the data (only to nutrients with an established RDA) to obtain approximate probability estimates of records within the distribution expected to reflect inadequate intakes below requirements. To apply this approach to the current data, assumptions were made about the distribution. Reasonable assumptions for most nutrients are that (a) the distribution of requirements is Gaussian in nature, (b) the coefficient of variation for one standard deviation (SD) is about 10%, and (c) there is a very low order of correlation between intake and requirement. It is further assumed that the RDA has been set at two SD above the mean requirement. In the absence of complete information, this appears to be a reasonable approximation, at least for data interpretation. At 20% (two SD) above the mean requirement, or the RDA, the probability of inadequate intake is 0.025. In contrast, for intakes 1 and 2 SD below the RDA, the probabilities of inadequate intake are 0.31 and 0.69, respectively. If it is assumed that the variance of the average intake is similar, then for intakes averaging 146% above the RDA and with a coefficient of variation of 10%, the risk of inadequate intake below the RDA is very low (0.07). This model allows us to estimate population-level risk of insufficient intake where usual intake distributions are not available. While this method is widely used, certain nutrients—such as vitamin D—may exhibit skewed distributions, which could affect the precision of inadequacy estimates.

The probability-based approach could not be applied to nutrients with only an Adequate Intake (AI), such as fiber, sodium, potassium, linoleic acid, and alpha-linolenic acid, due to the absence of defined requirement distributions.

### 2.5. Protein Quality Assessment

In addition to total protein intakes, the Protein Digestibility-Corrected Amino Acid Scores (PDCAASs) were used to adjust for the quality of protein intake in each group, as described elsewhere [18] and reported for adult diets [7]. PDCAAS was estimated using indispensable amino acid (IAA) concentrations of foods and an assumed digestibility coefficient of 0.8 and compared with a recommended IAA scoring pattern (mg/g protein requirement). The PDCAAS of the protein in diets was ≥0.86 for Groups 1−3, and 0.67 ± 0.027 in Group 4 (*p* < 0.0001). While the Digestible Indispensable Amino Acid Score (DIAAS) is recognized as a more refined method for evaluating protein quality—particularly for plant-based sources—DIAAS values were not available for the foods reported in the CCHS 2015 dataset. PDCAAS is also the regulatory standard for protein quality evaluation in Canada.

### 2.6. Statistical Analyses

Statistical analyses were conducted using the survey weights provided by Statistics Canada for the CCHS 2015 to ensure that findings are representative of the Canadian pediatric population. Variance estimation for the mean intake of foods was performed using the Balanced Repeated Replication (BRR) method with 500 replicate weights, as recommended by Statistics Canada [19]. Data preparation was completed using R Studio version 1.2.5019, and SAS version 9.4 (SAS Institute Inc., Cary, NC, USA) was used for all statistical analyses. Data for macro- and selected micronutrients were used to compare with the DRI or AI (Health Canada Reports 2023). Analysis of variance (ANOVA) by PROC SURVEYREG with the LSMEANS function compared nutrient intakes between the four groups. To satisfy normality, data were transformed to approximate a normal distribution using the Box-Cox method [20]. Groups 1, 3, and 4 were compared with the reference group (25–49.9%) using ADJUST SIMULATE. This post-hoc multiple comparison analysis reduces the risk of a false positive result in multiple comparisons. The significance level was set at *p* < 0.05 for differences between the reference group and the other 3 groups. Similar to our previous report on adults and the elderly [7] as well as others [4], the average intakes derived from this study were based on a single-day unadjusted 24 h dietary recall. Although a second 24 h dietary recall was collected from approximately 35% of participants to obtain an estimate of population variance, the public-use microdata file includes only the first recall, which included the total population. Therefore, this analysis was based on a single-day dietary recall for all children aged 9–18 years. Participants with missing data for key variables were excluded using listwise deletion, a common approach in large-scale dietary analyses. No imputation procedures were applied.

The reference group for statistical comparisons was selected because it contained the largest sample of children and, therefore, represented a potential quantitative target for Canada’s Food Guide, aligning with the advocacy for an increased consumption of plant-based foods.

## 3. Results

The characteristics of the study population in the four groups are summarized in Table 1. Records were derived from 2324 children aged 9 to 18 years. Groups 1, 2, 3, and 4 comprised 29.7%, 53.6%, 13.9%, and 2.7% of the sample, respectively. The largest number of records was in Group 2 (*n* = 1246), who reported deriving 25–49% of their protein intake from plant foods. Group 4 contained an inadequate number of records (*n* = 63) to provide confidence in the sample results; however, they are reported here. The demographics of the respondents in this study were similar across the plant protein groups, except for a higher body weight in females in Group 1, who were also the lowest consumers of plant protein foods (*p* < 0.05) (Table 1).

### 3.1. Nutrient Density by Protein Grouping

The proportions of energy derived from fat, protein, and carbohydrates in Group 1 were 34%, 20%, and 46%; in Group 2, they were 32%, 15%, and 54%; in Group 3, they were 29%, 13%, and 58%; and in Group 4, they were 5%, 11%, and 64%, respectively.

The nutrient density relative to energy intake (1000 kcal) in Groups 1, 3, and 4 was compared to the average (Group 2, 25–49.9% plant protein) (Table 2). Children in Group 1 (0–24.9% plant protein) had higher intakes than Group 2 of protein, fat, and saturated fatty acids (SFA), monounsaturated fatty acids (MUFA), cholesterol, vitamin D, potassium, vitamin B12, riboflavin, niacin, vitamin B6, phosphorus, and zinc (*p* < 0.0001), and lower intakes of carbohydrate and dietary fiber, folate, dietary folate equivalents (DFE), and thiamin (*p* < 0.0001). In Group 3 (50–74.9% plant protein), compared with Group 2, higher intakes of carbohydrate, dietary fiber, folate, DFE, iron, and magnesium (*p* < 0.001) were recorded. Lower intakes were reported for SFA, calcium, phosphorus, and B12. In Group 4, these differences from Group 2 were accentuated.

### 3.2. Energy and Nutrient Intakes by Age and Gender

#### 3.2.1. Females 9–13 Years

In 9- to 13-year-old females (Table 3), energy intake was similar across all four groups. For all females in Groups 1–3, folate and vitamin D intakes averaged below 60%, and calcium below 88%, of the RDA, reflecting probabilities of intakes below requirements of 0.69 and 0.93, respectively, based on being within the first SD below the mean requirement. The average intake for dietary fiber was less than 69% of the AI. Sodium intakes exceeded 220% of the recommended intake. The potassium average intake was close to the AI. There was no difference among groups in terms of dietary intakes of sodium, folate, linoleic and linolenic fatty acids, thiamin, riboflavin, and magnesium. In Group 1, protein, SFA, vitamin B12, vitamin B6, niacin, riboflavin, potassium, phosphorus, and zinc were highest, and fiber and iron were lowest. With increasingly plant-based diets, there were decreasing intakes of SFA, protein, vitamin D, calcium, vitamin B12, phosphorus, magnesium, and zinc. Average intakes of vitamin B12 were 41% below the RDA in Group 4 (Table 3).

While these patterns are statistically significant, several nutrients—including vitamin D, calcium, and vitamin B12—remained well below recommended levels across groups. This raises potential clinical concerns related to bone health and micronutrient adequacy in early adolescent females.

#### 3.2.2. Males 9–13 Years

As in females, energy intakes in 9–13-year-old males were similar across groups (Table 4). In Groups 1–3, folate and vitamin D intakes averaged below 67%, while calcium intakes averaged below 88% of the RDA, reflecting the probability of intakes below requirements. The average intake for dietary fiber was less than 69% of the AI. Sodium intakes also averaged over 250% of the recommended intake. Again, the potassium average intake was close to AI. There was no significant difference among Groups 1–3 in dietary intakes of calcium, iron, sodium, linoleic and linolenic fatty acids, vitamin B6, magnesium, or phosphorus. In Group 1, protein, SFA, vitamin B12, vitamin B6, niacin, riboflavin, potassium, phosphorus, and zinc were higher, and fiber and iron were lower compared to Group 2. With the increasing prevalence of plant-based diets, there were correspondingly lower intakes of SFA, protein, vitamin D, calcium, vitamin B12, phosphorus, and zinc. Average intakes of vitamin B12 were 24% below the RDA in Group 4 (Table 4).

#### 3.2.3. Females 14–18 Years

In 14–18-year-old females (Table 5), energy intake was similar in Groups 1–3 but lower in Group 4. Similar to the 9–13-year-old females in Groups 1–3, folate and vitamin D intakes averaged below <60%, and calcium and magnesium below <88%, of the RDA, reflecting probabilities of intakes below requirements of 0.69 and 0.93 in the first SD, respectively. The average intake for dietary fiber was less than 77% of the AI. Sodium intakes averaged over 170% of the recommended intake, but were markedly lower than 220% in the younger girls. The average intake of potassium was again close to AI. There was no difference between Groups 1–2 in dietary intakes of fat, protein, folate, calcium, sodium, iron, linoleic and linolenic fatty acids, thiamin, riboflavin, niacin, or magnesium. However, Group 1 was higher in vitamin D, B6, B12, phosphorus, potassium, and zinc, but lower in carbohydrates and fiber. In Group 3, compared with Group 2, protein, SFA, sodium, and vitamin B12 were lower. In Group 4, intakes of dietary fiber, iron, folate, and magnesium were highest, and vitamin D, niacin, riboflavin, B12, phosphorus, total fat, and SFA were lowest in this group compared with Group 2. With the increasing prevalence of plant-based diets, there were correspondingly lower intakes of SFA, protein, vitamin D, calcium, vitamin B12, phosphorus, and zinc. Average intakes of vitamin B12 were 50% below the RDA in Group 4 (Table 5).

#### 3.2.4. Males 14–18 Years (Table 6)

For 14–18-year-old males, energy intake was similar across all groups (Table 6). Similar to the 9–14-year-old females in Groups 1–3, folate and vitamin D intakes averaged below 60% of the RDA, reflecting the probability of intakes below requirements of 0.69 in the first SD. The average intake for dietary fiber was less than 77% of the AI. Sodium intakes averaged over 240%, similar to the 9–13-year-old boys. The average intake of potassium was close to the AI. There was no difference between Groups 1 and 2 in dietary intakes of fat, saturated fatty acids, folate, calcium, sodium, iron, linoleic and linolenic fatty acids, and thiamin. However, Group 1 was higher in protein, potassium, B12, riboflavin, niacin, vitamin B6, phosphorus, magnesium, and zinc, but lower in carbohydrates and fiber. In Group 3, compared with Group 2, most nutrient intakes were similar. Intakes in Group 4 compared with Group 2 were lower in total fat, protein, SFA, vitamin D, calcium, vitamin B12, vitamin B6, niacin, phosphorus, and zinc. Intakes of carbohydrates, dietary fiber, folate, thiamin, potassium, magnesium, and iron were higher in this group compared with Group 2. With the increasing prevalence of plant-based diets, there were correspondingly lower intakes of saturated fat, protein, vitamin D, calcium, vitamin B12, phosphorus, and zinc. Average intakes of vitamin B12 were 40% below the RDA in Group 4 (Table 6).

**Table 6:** Average nutrient intakes relative to plant protein intake compared with RDA and AI (%) for males (14–18 years) (*n* = 516).

Plant Protein/Total %	0–24.9% (*n* = 181)	25–49.9% (*n* = 270)	50–74.9% (*n* = 58)	75–100% (*n* = 7)	RDA AI
Total energy (kcal)	2479.44 ± 67.04	2369.28 ± 68.59	2490.8 ± 94.07	2384.35 ± 309.98	
Total fat (g)	92.92 ± 5.17	88.79 ± 4.21	82.6 ± 5.72	55.25 ± 13.44 *	ND
Total protein (g)	136 ± 9.08 *** (262%)	86.89 ± 3.02 (167%)	88.87 ± 7.57 (171%)	56.57 ± 13.53 (109%)	52
Total carbohydrate (g)	275.01 ± 10.88 * (212%)	311.87 ± 8.69 (240%)	353.82 ± 14.59 * (272%)	428.3 ± 91.37 (329%)	130
Total dietary fiber (g)	15.06 ± 0.84 *** (40%)	19.27 ± 0.87 (51%)	25.4 ± 1.71 ** (67%)	24.37 ± 7.83 (64%)	38
Total saturated fatty acids (g)	32.02 ± 2.09	30.1 ± 1.31	24.81 ± 1.99	16.11 ± 1.65 ***	As low as possible
Linoleic fatty acids (g)	15.24 ± 0.93 (95%)	15.29 ± 1.39 (96%)	16.87 ± 1.38 (105%)	12.42 ± 4.98 (78%)	16
Linolenic fatty acids (g)	1.79 ± 0.12 (112%)	1.78 ± 0.18 (111%)	1.7 ± 0.15 (106%)	1.95 ± 0.7 (122%)	1.6
Vitamin D (mcg)	8.34 ± 0.82 (56%)	5.94 ± 0.62 (40%)	5.82 ± 1.37 (39%)	2.45 ± 1.89 (16%)	15
Folate (mcg)	208.89 ± 13.38 (52%)	211.85 ± 7.43 (53%)	239.75 ± 36.42 (60%)	354.6 ± 150.51 (89%)	400
Vitamin B12 (mcg)	8.51 ± 1.08 ** (355%)	4.51 ± 0.31 (188%)	3.26 ± 0.46 (136%)	1.45 ± 0.74 * (60%)	2.4
Thiamin (mg)	2 ± 0.12 (167%)	2 ± 0.11 (167%)	2.83 ± 0.32 (236%)	2.7 ± 0.66 (225%)	1.2
Riboflavin (mg)	2.88 ± 0.33 * (222%)	2.18 ± 0.11 (168%)	2.38 ± 0.3 (183%)	1.99 ± 0.44 (153%)	1.3
Niacin (mg)	67.29 ± 6.01 *** (421%)	41.79 ± 1.43 (261%)	50.27 ± 6 (314%)	40.02 ± 10.16 (250%)	16
Vitamin B6 (mg)	3.13 ± 0.51 *** (241%)	1.72 ± 0.09 (132%)	1.93 ± 0.27 (148%)	1.52 ± 0.98 (117%)	1.3
Phosphorus (mg)	1956.44 ± 107.68 ** (157%)	1530.62 ± 54.71 (122%)	1546.66 ± 111.27 (124%)	1201.77 ± 269.95 (96%)	1250
Magnesium (mg)	366.77 ± 33.82 (89%)	317.94 ± 10.28 * (78%)	434.89 ± 46.39 * (106%)	340.3 ± 124.92 (83%)	410
Zinc (mg)	16.97 ± 1.14 ** (154%)	11.66 ± 0.53 (106%)	12.37 ± 1.27 (112%)	7.52 ± 1.97 (68%)	11
Calcium (mg)	1240.58 ± 69.62 (95%)	1115.52 ± 67.04 (86%)	1043.74 ± 99.06 (80%)	783.44 ± 196.3 (60%)	1300
Iron (mg)	16.16 ± 1.4 (147%)	15.48 ± 0.69 (141%)	20.07 ± 1.93 (182%)	16.02 ± 3.47 (146%)	11
Sodium (mg)	3706.14 ± 222.95 (247%)	3558.97 ± 210.45 (237%)	3490.21 ± 173.58 (233%)	3624.96 ± 719.27 (242%)	1500
Potassium (mg)	3324.9 ± 121.42 * (111%)	2847.72 ± 110.26 (95%)	3145.88 ± 226.31 (105%)	2958.59 ± 1612.81 (99%)	3000

Mean values significantly different from the 25–49.9% group (* *p* < 0.05; ** *p* < 0.01; *** *p* < 0.0001). Values are presented as mean ± standard error (SE). Values in parentheses represent the percentage of the recommended intake met by each group, based on the Recommended Dietary Allowance (RDA) or the adequate intake (AI), as appropriate. Comparisons were made using ANOVA with post-hoc simulation adjustment. Energy recommendations are based on the estimated energy requirements for moderately active Canadian children. Note: Zero males belong to the 0% animal protein source category.

### 3.3. Protein Sources and Quality

**Figure 1:**
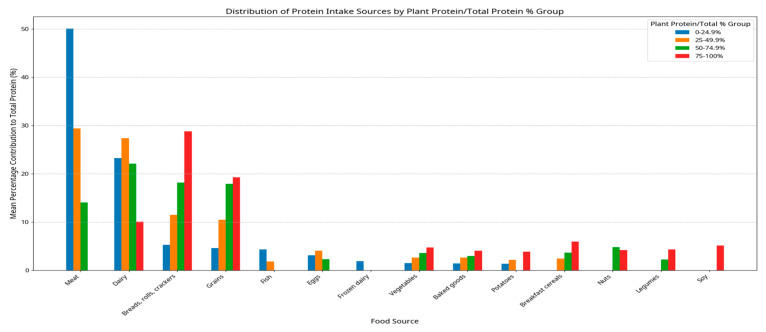
Distribution of protein intake sources by percentage of plant protein contribution to total protein intake in each group in children 9–18 years old. Group 1: 0–24.9% plant protein. Group 2: 24–49.9%. Group 3: 50–74.9%. Group 4: 75–100%. Data according to a single 24 h dietary recall from the Canadian Community Health Survey (CCHS) 2015, based on data from Canadian children and adolescents aged 9–18 years.

Protein intake in all children averaged 64.2% animal and 35.8% plant protein (Table 7). The top 10 foods consumed within each of the plant protein groupings are shown for all children (9 to 18 years) in Figure 1. Meat and dairy were the top two consumed sources of protein in the first two groups. Dairy was a top-three protein source across all four groups. In the fourth group, where plant protein contributed >75% of daily protein intake, breads, rolls, crackers, grains, dairy, and breakfast cereals were the top sources. Based on the major food sources contributing to plant protein intake in this cohort, ultra-processed plant-based meat analogues did not feature prominently among the top contributors, and were therefore presumed to constitute only a minimal fraction of total plant protein intake during the CCHS 2015 survey period.

Protein decreased with increased plant protein intakes (Table 8). Total day protein consumption decreased within the combined sex and age data in groups with increasing intakes of plant protein sources, from 106.12 ± 3.46 g/day in Group 1 to 50.07 ± 3.95 g/day in Group 4. Similar trends were demonstrated when protein intakes were expressed relative to body weight and energy intake. Across all groups, absolute protein intakes averaged >0.8 g/kg body weight.

The PDCAAS for total protein intake was the highest in Group 1 (0.99  ±  0.002) and decreased as plant protein consumption increased to a low in Group 4 of 0.67 ± 0.027 (Table 8). Although PDCAAS values in Groups 2 and 3 remained above 0.85, there was a reduction in corrected protein compared to absolute protein in the groups where plant protein consumption was greater (reductions were ~3 g in Group 2, ~10 g in Group 3, and ~18 g in Group 4). These trends were also apparent when corrected protein was expressed relative to body weight as well as percent energy from corrected protein. Both measures decreased as plant protein consumption increased. Reductions from Group 1 to Group 4 were more than two-fold in corrected protein relative to body weight (1.99 ± 0.05 vs. 0.68 ± 0.06) as well as percent energy from corrected protein (20.07 ± 0.69 vs. 7.23 ± 0.66) (Table 8).

## 4. Discussion

This analysis of nutrient intakes in Canadian children aged 9–18 years provides insights into the nutritional consequences of increasing the ratio of plant to animal protein in their diets. The results support the CFG recommendation to increase the proportion of plant foods in their diets, and highlight the importance of a balanced approach to the inclusion of animal and plant protein sources. Furthermore, the results support previous conclusions that Canadian children are at risk of not meeting their requirements for certain nutrients, including calcium, potassium, and vitamin D, as well as fiber intake, and they also consume excess sodium.

The proportions of energy from fat, protein, and carbohydrates in Group 3 were 30%, 13%, and 59%, as is consistent with dietary guidelines [21]. Similarly, the average nutrient intakes of Groups 1–3 were adequate to meet the recommended amounts for most children. However, in Groups 1–3, the estimated average intakes of vitamin D and folate were below 66%, and that for calcium was below 88%, of the RDA, thus predicting probabilities of inadequate intakes of 0.93 and 0.31. The analysis of age and sex showed that the majority of nutrient intakes were similar and approximated acceptable intakes across Groups 1–3, but were also affected by sex and age.

In this population of 9–18-year-old children, the average diet reported on any given day consisted of 64% animal and 36% plant protein, similar to dietary patterns reported in adults [7,18]. In addition, similar patterns of consumption were reported in the US [22,23] and Europe [24], and the quantity and quality of protein intake met the recommended standards except in Group 4. However, the records reporting 75% or more of dietary protein from plant sources are very small (less than 4%), and caution is required in interpreting the data to derive public health action due to the large variance of the mean.

Others have reported that only 5% of the Canadian population follows a plant-based dietary pattern [25]. While these numbers may increase in response to the CFG [5] and with the significantly greater availability of plant-based foods and animal protein substitutes on the market today, the current evaluation supports a recent recommendation that strategies are needed to prevent nutritional inadequacies [8]. Furthermore, there is evidence that 3–5-year-old children who derive approximately 45% of their diet from ultra-processed foods, many as substitutes for animal protein foods, are obese [26]. The inadequate intakes of dietary fiber and potassium and excess intakes of sodium in school-aged children are consistent with previous reports [3,27]. Unlike in adults [7], a decrease in sodium intake with increasing plant protein consumption in children was not found. Salty snacks are the primary grain-based food, contributing nearly one-fifth of total energy intake [28]. Where plant protein intakes were less than 50% of the total protein (groups 1 and 2), meat, dairy, breads, rolls, and crackers contributed 70–75% of the total daily protein (Table 8), similar to that of Spanish children [29]. As the contribution of plant protein to total protein increased beyond 50% (Groups 3 and 4), breads, rolls and crackers, as well as dairy, along with grains, were the top protein sources, similar to Canadian adults [7]. Breakfast cereals, grains, and loaves of bread contributed most of the plant protein, sourced from refined starches, with little consumption of whole-grain foods [8]. In Canadian children, refined/enriched grains rather than whole-grain foods are consumed [30].

Dairy foods were among the top three protein sources across all four groups. Both vitamin D and calcium intakes were inadequate, but vitamin D from sun exposure and supplements is not reported. However, based on blood vitamin D (50 nmol/L), 79% and 68% of children in 2007/2009 and 2012/2013, respectively, were vitamin D-sufficient. Those at risk are found in sun-exposure regions and those with dark skin, and these people require focused support [31]. However, the less-than-desired calcium intake indicates a need for recommendations for children to increase dairy intake, as are found in previous food guides. Dairy products promote bone and cardiometabolic health in children [32,33,34,35]. Health Canada’s recent marketing authorization will enable manufacturers to voluntarily increase the vitamin D levels in cow’s milk, goat’s milk, and margarine [36]. It remains to be seen if this will reduce clinically relevant measures of deficiency in children.

The decline in milk consumption [37] may be explained by several factors. Many consumers are shifting towards plant-based beverages such as almond beverages. Additionally, Health Canada has fortified soy beverages as a source of protein [5,37]. However, except for soy beverages, all other plant-based products are typically lower in both content and quality compared to dairy milk [38]. Children in Toronto who consume cow’s milk substitutes were reported to be shorter than those who consume cow’s milk [39]. More recently, lower zBMI (BMI z-score) was associated with an increasing intake of full-fat cow’s milk in children aged 9 months to 8 years. Children who consumed whole milk had a 16% lower risk of being overweight and an 18% lower risk of obesity compared to those who consumed low-fat milk [40].

The adequacy of protein intake for most children, except for those on vegan diets (Group 4), indicates that the protein quality labeling of foods in Canada may not be a priority. Instead, strategies should focus on increasing food literacy in families of young children to create a better understanding of the protein category of the CFG, as recommended by a recent expert and stakeholder workshop [41]. The CCHS 2015 offers valuable population-level dietary data, and its methodological strengths include the survey design, which aimed for balanced seasonal and daily data collection, although formal temporal adjustments were not applied in our analyses. Additionally, the reliance on a single 24 h recall standard for such surveys does not capture habitual intake due to day-to-day dietary variations. Thus, the assumption of a normal distribution for all nutrient intakes in the probability method may not fully capture the variability of certain nutrients, such as vitamin D, which often exhibit skewed distributions.

Although the 2019 Canada’s Food Guide encourages the increased consumption of plant-based protein sources due to their health and environmental benefits, our findings highlight the importance of considering potential nutritional trade-offs, especially in vulnerable populations such as children. A shift towards predominantly plant-based diets, if not carefully planned, may inadvertently increase the risk of inadequate intakes of key micronutrients such as iron, zinc, and vitamin B12—nutrients that are more bioavailable in animal-based foods. These risks underscore the need for appropriate strategies such as food fortification and supplementation. Public health messaging must therefore strike a balance between promoting plant-forward eating patterns and ensuring nutritional adequacy, particularly in pediatric populations. From a public health perspective, these findings suggest that while promoting plant-based dietary patterns remains a valuable strategy, a more cautious approach is warranted—particularly for children and other vulnerable groups. Ensuring the adequate intake of all essential nutrients may require the systematic use of fortified foods, targeted supplementation (e.g., vitamin B12, iron, and calcium), and comprehensive dietary education. These measures are critical to preventing the emergence or exacerbation of nutrient deficiencies as plant-forward dietary trends continue to gain traction.

This study has several strengths, including the use of nationally representative dietary intake data from the 2015 CCHS—Nutrition, and the application of a probability approach to estimate the potential risk of nutrient inadequacy based on the group-level estimates of nutrient intake and the assumption of a normal distribution. However, the study also has limitations. The use of probability analysis was limited by the lack of estimates of individual habitual intakes. Additionally, our study did not differentiate among plant protein sources based on their degree of processing (e.g., whole versus ultra-processed plant-based foods), and it is recognized that nutrient profiles can vary significantly with processing levels. While these products were likely infrequently consumed during the CCHS 2015 survey period, their increasing prevalence in recent years highlights the importance of this distinction as an avenue for future investigation [42]. Finally, the findings are specific to the Canadian population and may not be generalizable to other populations with different food availability and cultural contexts. In summary, this study highlights the importance of implementing supportive strategies to ensure nutrient adequacy when transitioning to more plant-based diets in children, as shown in the present study. As dietary patterns continue to evolve in response to health, environmental, and ethical considerations, the ongoing monitoring of nutrient adequacy in children’s diets will be essential to ensure that nutritional needs are met during this critical period of growth and development. Future public health initiatives should focus on increasing food literacy among families with young children, emphasizing the importance of dietary balance and nutrient quality, rather than simply the protein source.

## 5. Conclusions

A protein intake pattern characterized by a 3:1 ratio of animal-to-plant protein was associated with a favorable macronutrient distribution and adequate intakes of most essential nutrients. However, Canadian children may be at risk of developing nutrient inadequacies when the majority of protein is derived from plant-based foods, particularly without attention to dietary diversity and nutrient density. 

## Figures and Tables

**Table 1:** Participant characteristics by percentage of plant protein in total daily protein intake.

	Plant Protein Groups ^1^			
	0–24.9%	25–49.9%	50–74.9%	75–100%
	All Children (*n* = 2324)			
N	691 (29.73%)	1246 (53.61%)	324 (13.94%)	63 (2.71%)
Weight (kg)	55.74 ± 1.24	54.84 ± 1.02	55.62 ± 2.16	50.37 ± 5.19
Height (m)	1.6 ± 0.01	1.58 ± 0.01	1.59 ± 0.02	1.58 ± 0.03
BMI	21. 42 ± 0.29	21.47 ± 0.27	21.55 ± 0.47	20.02 ± 1.54
Age (y)	13.58 ± 0.17	13.06± 0.13	13.3± 0.29	13.55± 0.66
	Females (*n* = 1151)			
N	300 (26.06%)	623 (54.13%)	183 (15.90%)	45 (3.91%)
Weight (kg)	53.72 ± 1.37 *	51.64 ± 1	51.98 ± 1.82	50.06 ± 6.72
Height (m)	1.57 ± 0.01	1.55 ± 0.01	1.56 ± 0.01	1.56 ± 0.03
BMI	21.59 ± 0.41	21.12 ± 0.34	21.08 ± 0.56	20. 24 ± 2.21
Age (y)	13.75 ± 0.23	12.92 ± 0.16	13.36 ± 0.3	13.83 ± 0.8
	Males (*n* = 1173)			
N	391 (33.33%)	623 (53.11%)	141 (12.02%)	18 (1.53%)
Weight (kg)	57.43 ± 1.86	57.56 ± 1.63	59.92 ± 4.7	51.19 ± 6.99
Height (m)	1.62 ± 0.01	1.6 ± 0.01	1.62 ± 0.03	1.61 ± 0.07
BMI ^2^	21.28 ± 0.41	21.77 ± 0.43	22.10 ± 0.96	19.42 ± 1.31
Age (y)	13.44 ± 0.26	13.18 ± 0.19	13.23 ± 0.53	12.82 ± 1.31

Mean values significantly different from the 25–49.9% group (* *p* < 0.05). Values are presented as mean ± standard error (SD). ^1^ Plant protein (%) in the total daily protein intake. ^2^ BMI: Body Mass Index.

**Table 2:** Nutrient density (per 1000 kcal) in all children (9–18 years) by percentage of plant protein intake (*n* = 2324).

Plant Protein/Total %	0–24.9% (*n* = 691)	25–49.9% (*n* = 1246)	50–74.9% (*n* = 324)	75–100% (*n* = 63)
Total fat (g)	37.93 ± 0.62 **	35.61 ± 0.38	32.77 ± 0.76 **	28.4 ± 1.83 **
Total protein (g)	51.1 ± 1.72 ***	37.14 ± 0.37	31.9 ± 1.23 **	27.98 ± 1.5 ***
Total carbohydrate (g)	114.69 ± 2.01 ***	135.66 ± 0.93	148.31 ± 1.69 ***	163.65 ± 4.1 ***
Total dietary fiber (g)	6.43 ± 0.18 ***	8.49 ± 0.15	10.53 ± 0.26 ***	13.26 ± 0.85 ***
Total saturated fatty acids (g)	14.08 ± 0.32 **	12.58 ± 0.17	10.24 ± 0.29 ***	8.51 ± 0.62 ***
Cholesterol (mg)	173.83 ± 5.59 ***	121.42 ± 4.35	64.81 ± 4.83 ***	36.76 ± 6.49 ***
MUFA (g)	13.85 ± 0.3 *	12.87 ± 0.17	12.06 ± 0.46	9.63 ± 0.93 **
PUFA (g)	6.66 ± 0.17	6.95 ± 0.19	7.34 ± 0.24	7.06 ± 0.83
Linoleic fatty acids (g)	5.56 ± 0.16	5.97 ± 0.18	6.46 ± 0.22	6.11 ± 0.76
Linolenic fatty acids (g)	0.7 ± 0.02	0.73 ± 0.03	0.74 ± 0.03	0.79 ± 0.09
Vitamin D (mcg)	3.71 ± 0.24 **	2.81 ± 0.1	2.33 ± 0.26	1.55 ± 0.22 ***
Folate (mcg)	82.61 ± 2.71 **	94.11 ± 1.99	101.26 ± 6.89	128.74 ± 11.13 *
Folic acid (mcg)	50.75 ± 4.85 ***	76.85 ± 2.43	99.92 ± 7.14 *	102.55 ± 10.52
Dietary folate equivalents (mcg)	183.59 ± 9.98 ***	250.01 ± 4.11	297.64 ± 10.2 ***	328.42 ± 26.74 **
Folacin (mcg)	133.58 ± 6.72 ***	170.59 ± 2.41	201.32 ± 6.72 **	231.63 ± 12.94 ***
Vitamin B12 (mcg)	3.29 ± 0.23 ***	2.21 ± 0.29	1.25 ± 0.09 ***	0.68 ± 0.09 ***
Thiamin (mg)	0.81 ± 0.02 **	0.9 ± 0.02	1.01 ± 0.05	1.27 ± 0.11**
Riboflavin (mg)	1.11 ± 0.04 **	0.99 ± 0.02	0.89 ± 0.04	0.8 ± 0.05**
Niacin (mg)	23.74 ± 0.99 ***	17.52 ± 0.22	16.72 ± 0.66	16.03 ± 0.82
Vitamin B6 (mg)	1.01 ± 0.06 ***	0.74 ± 0.02	0.71 ± 0.03	0.75 ± 0.07
Phosphorus (mg)	772.93 ± 18.36 ***	671.76 ± 7.36	618.67 ± 21.35 *	539.61 ± 33.4**
Magnesium (mg)	138.66 ± 5.27	137.83 ± 1.72	156.42 ± 6.15 *	167.45 ± 10.29*
Zinc (mg)	6.73 ± 0.34 ***	4.9 ± 0.08	4.41 ± 0.19	3.84 ± 0.2 ***
Calcium (mg)	522.6 ± 19.1	512.21 ± 9.63	446.78 ± 20.54 **	372.56 ± 40.69**
Iron (mg)	6.14 ± 0.24 ***	6.88 ± 0.1	7.6 ± 0.27 *	7.95 ± 0.45*
Sodium (mg)	1450.65 ± 40.61	1477.51 ± 24.18	1389.12 ± 30.16	1328.79 ± 77.08
Potassium (mg)	1377.01 ± 22.57 **	1274.95 ± 21.22	1225.47 ± 38.81	1258.76 ± 127.09

Mean values significantly different from the 25–49.9% group (* *p* < 0.05; ** *p* < 0.01; *** *p* < 0.0001). Values are presented as mean ± standard error (SE). Comparisons were made using ANOVA with post-hoc simulation adjustment. Note: Five children (0.21%) belonged to the 0% animal protein source category.

**Table 3:** Average nutrient intakes related to plant protein intake compared with the RDA or AI (%) for females (9–13 years) (*n* = 609).

Plant Protein/Total %	0–24.9% (*n* = 146)	25–49.9% (*n* = 350)	50–74.9% (*n* = 97)	75–100% (*n* = 16)	RDA AI
Total energy (kcal)	1919.7 ± 37.04	1878.03 ± 54.76	1816.85 ± 48.07	1765.69 ± 138.18	
Total fat (g)	71.93 ± 2.64	64.9 ± 2.38	56.35 ± 2.73	47.67 ± 8.82	ND
Total protein (g)	93.25 ± 4.22 *** (274%)	67.78 ± 2.05 (199%)	52.58 ± 2.1 *** (155%)	49.71 ± 7.42 (146%)	34
Total carbohydrate (g)	228.5 ± 8.95 ** (176%)	261.46 ± 8.14 (201%)	281.73 ± 9.37 (217%)	291.49 ± 32.34 (224%)	130
Total dietary fiber (g)	12.93 ± 0.8 ** (50%)	16.19 ± 0.63 (62%)	17.69 ± 0.99 (68%)	17.74 ± 2.2 (68%)	26
Total saturated fatty acids (g)	27.56 ± 1.31 *	23.43 ± 0.85	18.57 ± 1.12 **	15.02 ± 2.58 *	As low as possible
Linoleic fatty acids (g)	9.87 ± 0.79 (99%)	10.76 ± 0.58 (108%)	10.93 ± 0.72 (109%)	9.29 ± 2.78 (93%)	10
Linolenic fatty acids (g)	1.31 ± 0.1 (131%)	1.34 ± 0.06 (134%)	1.43 ± 0.14 (143%)	1.09 ± 0.31 (109%)	1
Vitamin D (mcg)	7.14 ± 1.04 (48%)	5.42 ± 0.25 (36%)	4.51 ± 0.66 (30%)	2.21 ± 0.61 ** (15%)	15
Folate equivalents(mcg)	170.82 ± 9.44 (57%)	182.78 ± 10.44 (61%)	165.06 ± 13.11 (55%)	212.26 ± 57.32 (71%)	300
Vitamin B12 (mcg)	5.54 ± 0.45 ** (308%)	5.32 ± 1.67 (296%)	2.04 ± 0.2 *** (113%)	1.06 ± 0.24 *** (59%)	1.8
Thiamin (mg)	1.6 ± 0.1 (178%)	1.72 ± 0.07 (191%)	1.95 ± 0.19 (217%)	1.95 ± 0.26 (217%)	0.9
Riboflavin (mg)	2.1 ± 0.09 (233%)	1.91 ± 0.11 (212%)	1.61 ± 0.11 (179%)	1.4 ± 0.24 (156%)	0.9
Niacin Equivalents (mg)	42.04 ± 2.56 *** (350%)	31.59 ± 1.08 (263%)	27.78 ± 1.34 (232%)	28.19 ± 2.6 (235%)	12
Vitamin B6 (mg)	1.74 ± 0.09 ** (174%)	1.34 ± 0.05 (134%)	1.15 ± 0.09 (115%)	1.01 ± 0.2 (101%)	1.0
Phosphorus (mg)	1476.75 ± 56.15* (118%)	1252.3 ± 37.14 (100%)	1017.66 ± 54.28** (81%)	835.56 ± 128.6 * (67%)	1250
Magnesium (mg)	260.3 ± 7.45 (108%)	256.89 ± 7.26 (107%)	245.08 ± 12.49 (102%)	234.44 ± 22.5 (98%)	240
Zinc (mg)	11.71 ± 0.68 *** (146%)	9.06 ± 0.32 (113%)	7 ± 0.31 *** (88%)	5.92 ± 0.84 * (74%)	8.0
Calcium (mg)	1040.39 ± 82.36 (80%)	985.14 ± 41.27 (76%)	822.08 ± 100.72 (63%)	542.93 ± 139.08 * (42%)	1300
Iron (mg)	11.86 ± 0.41 ** (148%)	13.85 ± 0.42 (173%)	13.92 ± 0.55 (174%)	14.1 ± 0.99 (176%)	8
Sodium (mg)	2696.09 ± 124.01 (225%)	2644.24 ± 93.01 (220%)	2583.63 ± 125.65 (215%)	2300.3 ± 295.81 (192%)	1200
Potassium (mg)	2745.27 ± 89.76 ** (119%)	2438.86 ± 91.87 (106%)	2144.69 ± 166 (93%)	1964.31 ± 269.35 (85%)	2300

Mean values significantly different from the 25–49.9% group (* *p* < 0.05; ** *p* < 0.01; *** *p* < 0.0001). Values are presented as mean ± standard error (SE). Values in parentheses represent the percentage of the recommended intake met by each group, based on the Recommended Dietary Allowance (RDA) or the adequate intake (AI), as appropriate. Comparisons were made using ANOVA with post-hoc simulation adjustment. Energy recommendations are based on the estimated energy requirements for moderately active Canadian children.

**Table 4:** Average nutrient intakes by plant protein intake compared with RDA and AI (%) for males (9–13 years) (*n* = 657).

Plant Protein/Total%	0–24.9% (*n* = 210)	25–49.9% (*n* = 353)	50–74.9% (*n* = 83)	75–100% (*n* = 11)	RDA AI
Total energy (kcal)	2055.53 ± 49.24	2022.08 ± 47.28	1870.76 ± 56.86	2086.8 ± 185.15	
Total fat (g)	79.45 ± 2.62	71.55 ± 2.12	62.57 ± 4.6	66.54 ± 7.69	ND
Total protein (g)	95.79 ± 4.47 ** (282%)	75.47 ± 2.23 (222%)	59.67 ± 3.37 ** (176%)	49.37 ± 6.45 ** (145%)	34
Total carbohydrate (g)	243.61 ± 8.59 ** (187%)	275.14 ± 6.68 (212%)	274.62 ± 9.19 (211%)	332.17 ± 30.65 (256%)	130
Total dietary fiber (g)	13.62 ± 0.8 ** (44%)	17.47 ± 0.74 (56%)	21.07 ± 1.34 * (68%)	28.18 ± 6.76 (91%)	31
Total saturated fatty acids (g)	30.67 ± 1.31 **	25.49 ± 0.99	20.49 ± 1.09 *	17.07 ± 2.91	As low as possible
Linoleic fatty acids (g)	10.95 ± 0.74 (91%)	11.83 ± 0.44 (99%)	12.08 ± 1.46 (101%)	13.83 ± 2.19 (115%)	12
Linolenic fatty acids (g)	1.35 ± 0.07 (113%)	1.5 ± 0.08 (125%)	1.53 ± 0.16 (128%)	1.3 ± 0.2 (108%)	1.2
Vitamin D (mcg)	6.55 ± 0.6 (44%)	5.74 ± 0.38 (38%)	4.13 ± 0.48 (28%)	4.07 ± 1.22 (27%)	15
Folate (mcg)	154.08 ± 9.3 * (51%)	185.41 ± 7.8 (62%)	199.39 ± 21.83 (66%)	210.34 ± 38.88 (70%)	300
Vitamin B12 (mcg)	5.95 ± 0.35 *** (331%)	3.85 ± 0.19 (214%)	2.43 ± 0.25 *** (135%)	1.36 ± 0.51 ** (76%)	1.8
Thiamin (mg)	1.57 ± 0.1 * (174%)	1.85 ± 0.06 (206%)	1.78 ± 0.16 (198%)	2.78 ± 0.43 * (309%)	0.9
Riboflavin (mg)	2.2 ± 0.1 (244%)	2.01 ± 0.07 (223%)	1.56 ± 0.07 *** (173%)	1.73 ± 0.31 (192%)	0.9
Niacin (mg)	42.95 ± 1.98 * (358%)	35.46 ± 0.98 (296%)	29.38 ± 1.29 ** (245%)	31.36 ± 4.57 (261%)	12
Vitamin B6 (mg)	1.73 ± 0.09 (173%)	1.51 ± 0.05 (151%)	1.25 ± 0.1 (125%)	1.77 ± 0.27 (177%)	1.0
Phosphorus (mg)	1506.03 ± 51.02 (120%)	1366.13 ± 40.28 (109%)	1223 ± 51.2 (98%)	1104.14 ± 151.52 (88%)	1250
Magnesium (mg)	264.26 ± 11.26 (110%)	277.22 ± 7.72 (116%)	280.34 ± 13.55 (117%)	348.99 ± 52.12 (145%)	240
Zinc (mg)	13.34 ± 0.94 ** (167%)	9.93 ± 0.34 (124%)	8.31 ± 0.44 (104%)	7.61 ± 1.25 (95%)	8.0*
Calcium (mg)	1091.97 ± 49.88 (84%)	1043.29 ± 40.21 (80%)	902.89 ± 65 (69%)	591.26 ± 90.62 *** (45%)	1300
Iron (mg)	12.58 ± 0.64 (157%)	14.39 ± 0.48 (180%)	14.24 ± 0.89 (178%)	15.24 ± 1.83 (190%)	8
Sodium (mg)	3003.33 ± 145.77 (250%)	3069.49 ± 98.86 (256%)	2731.4 ± 114.66 (228%)	2576.68 ± 225.74 (215%)	1200
Potassium (mg)	2752.18 ± 101.77 (110%)	2629.57 ± 82.74 (105%)	2233.12 ± 153.28 (89%)	2595 ± 360.22 (104%)	2500

Mean values significantly different from the 25–49.9% group (* *p* < 0.05; ** *p* < 0.01; *** *p* < 0.0001). Values are presented as mean ± standard error (SE). Values in parentheses represent the percentage of the recommended intake met by each group, based on the Recommended Dietary Allowance (RDA) or the adequate intake (AI), as appropriate. Comparisons were made using ANOVA with post-hoc simulation adjustment. Energy recommendations are based on the estimated energy requirements for moderately active Canadian children. Note: One child (0.15%) belongs to the 0% animal protein source category.

**Table 5:** Average nutrient intakes by plant protein intake compared with RDA and AI (%) for females (14–18 years) (*n* = 542).

Plant Protein/Total %	0–24.9% (*n* = 154)	25–49.9% (*n* = 273)	50–74.9% (*n* = 86)	75–100% (*n* = 29)	RDA AI
Total energy (kcal)	1882.86 ± 62.56	1836.29 ± 29.25	1823.57 ± 53.93	1637.49 ± 53.8 *	
Total fat (g)	74.83 ± 3.68	66.67 ± 1.67	62.25 ± 3.46	47.74 ± 3.46 **	ND
Total protein (g)	95.5 ± 6.07 (208%)	69.97 ± 1.85 (152%)	56.33 ± 2.95 ** (122%)	48.81 ± 4.23 *** (106%)	46
Total carbohydrate (g)	207.97 ± 11.14 ** (160%)	244.41 ± 4.59 (188%)	269.09 ± 9.54 * (207%)	263.54 ± 13.02 (203%)	130
Total dietary fiber (g)	11.55 ± 0.91 ** (44%)	15.19 ± 0.85 (58%)	19.49 ± 1.19 * (75%)	25.97 ± 2.92 ** (100%)	26
Total saturated fatty acids (g)	28.16 ± 1.82	23.75 ± 0.84	18.32 ± 1.27 *	14.6 ± 1.61 ***	As low as possible
Linoleic fatty acids (g)	10.92 ± 0.7 (99%)	11.29 ± 0.47 (103%)	12.37 ± 0.98 (112%)	10.91 ± 2.24 (99%)	11
Linolenic fatty acids (g)	1.44 ± 0.11 (131%)	1.39 ± 0.08 (126%)	1.24 ± 0.08 (113%)	1.46 ± 0.28 (133%)	1.1
Vitamin D (mcg)	8.42 ± 1.12 ** (56%)	4.95 ± 0.3 (33%)	4.04 ± 0.86 (27%)	2.79 ± 0.6 * (19%)	15
Folate (mcg)	153.35 ± 9.01 (38%)	176.24 ± 7.93 (44%)	200.95 ± 27.01 (50%)	220.72 ± 20.6 (55%)	400
Vitamin B12 (mcg)	6.76 ± 1.01 ** (282%)	3.37 ± 0.15 (140%)	2.22 ± 0.36 * (93%)	1.2 ± 0.3 ** (50%)	2.4
Thiamin (mg)	1.47 ± 0.1 (147%)	1.6 ± 0.08 (160%)	1.59 ± 0.1 (159%)	2.24 ± 0.33 (224%)	1.0
Riboflavin (mg)	2.01 ± 0.11 (201%)	1.8 ± 0.05 (180%)	1.61 ± 0.14 (161%)	1.26 ± 0.1 ** (126%)	1.0
Niacin (mg)	43.48 ± 3.23 (311%)	32.85 ± 1.01 (235%)	28.41 ± 1.22 * (203%)	25.74 ± 1.64 ** (184%)	14
Vitamin B6 (mg)	1.75 ± 0.12 * (146%)	1.32 ± 0.09 (110%)	1.31 ± 0.11 (109%)	1.41 ± 0.13 (118%)	1.2
Phosphorus (mg)	1438.73 ± 59.32 * (115%)	1246.94 ± 33.94 (100%)	1160.88 ± 100.49 (93%)	958.56 ± 85.43 * (77%)	1250
Magnesium (mg)	251.5 ± 9.08 (70%)	255 ± 10.45 (71%)	299.57 ± 21.91 (83%)	311.95 ± 22.83 * (87%)	360
Zinc (mg)	12.77 ± 1.35 * (142%)	8.96 ± 0.39 (100%)	7.76 ± 0.43 (86%)	7.11 ± 0.58 (79%)	9
Calcium (mg)	972.51 ± 88.83 (75%)	945.08 ± 35.09 (73%)	805.35 ± 92.1 (62%)	726.1 ± 120.05 (56%)	1300
Iron (mg)	10.78 ± 0.5 (72%)	11.75 ± 0.49 (78%)	13.02 ± 0.78 (87%)	14.11 ± 1.31 * (94%)	15
Sodium (mg)	2688.5 ± 190.71 (179%)	2702.66 ± 67.29 (180%)	2267.82 ± 107.39 ** (151%)	2140.68 ± 257.74 (143%)	1500
Potassium (mg)	2555.75 ± 93.39 * (111%)	2269.72 ± 77.24 (99%)	2254.66 ± 151.48 (98%)	2181.56 ± 234.46 (95%)	2300

Mean values significantly different from the 25–49.9% group (* *p* < 0.05; ** *p* < 0.01; *** *p* < 0.0001). Values are presented as mean ± standard error (SE). Values in parentheses represent the percentage of the recommended intake met by each group, based on the Recommended Dietary Allowance (RDA) or the adequate intake (AI), as appropriate. Comparisons were made using ANOVA with post-hoc simulation adjustment. Energy recommendations are based on the estimated energy requirements for moderately active Canadian children. Note: Four females (0.74%) belong to the 0% animal protein source category.

**Table 7:** Average proportion of protein sources in Canadian children (%).

Protein Source	All Children (*n* = 2324)	Females (*n* = 1151)	Males (*n* = 1173)
Animal protein (%)	64.20 ± 0.71	62.75 ± 1.02	65.53 ± 0.87
Plant protein (%)	35.80 ± 0.71	37.25 ± 1.02	34.47 ± 0.87

Values are presented as mean ± standard error (SE).

**Table 8:** Energy and protein intakes (total and corrected) by plant protein intakes in all children (9–18 years).

	Plant Protein Group ^1^				
	0–24.9% (*n* = 691)	25–49.9% (*n* = 1246)	50–74.9% (*n* = 324)	75–100% (*n* = 63)	Requirements (RDA)
PDCAAS	0.99 ± 0.002 ***	0.97 ± 0.003	0.86 ± 0.008 ***	0.67 ± 0.027 ***	~1
Total protein (g)	106.12 ± 3.46 ***	75.29 ± 1.12	63.68 ± 3.46 **	50.07 ± 3.95 ***	range: 34–52, depending on age/sex
Energy from protein (kcal)	20.37 ± 0.71 ***	14.69 ± 0.15	12.54 ± 0.49 **	10.94 ± 0.57 ***	10–30% of total energy intake
Protein by weight (g/kg)	2.02 ± 0.05 ***	1.52 ± 0.03	1.24 ± 0.04 ***	1.06 ± 0.08 ***	range: 0.85–0.95, depending on age group
Corrected protein by weight (g/kg)	1.99 ± 0.05 ***	1.47 ± 0.03	1.03 ± 0.04 ***	0.68 ± 0.06 ***	range: 0.85–0.95, depending on age group
Corrected protein (g)	104.09 ± 3.41 ***	72.34 ± 1.12	53.37 ± 3.17 ***	32.16 ± 3.64 ***	range: 34–52, depending on age/sex
Energy from corrected protein (kcal)	20.07 ± 0.69 ***	14.28 ± 0.16	10.71 ± 0.48 ***	7.23 ± 0.66 ***	10–30% of total energy intake

¹ Groups categorized by plant protein contribution to total protein intake. Mean values significantly different from the 25–49.9% group (** *p* < 0.01; *** *p* < 0.0001). Values are presented as mean ± standard error (SE). Comparisons were made using ANOVA with post-hoc simulation adjustment.

## Data Availability

Publicly available data from the Canadian Community Health Survey (CCHS) 2015—Nutrition were analyzed in this study.
